# Redefining the treatment of Chagas disease: a review of recent clinical and pharmacological data for a novel formulation of nifurtimox

**DOI:** 10.1371/journal.pntd.0012849

**Published:** 2025-02-25

**Authors:** Jaime Altcheh, Ulrike Grossmann, Heino Stass, Martin Springsklee, Facundo Garcia-Bournissen

**Affiliations:** 1 Hospital de Niños Ricardo Gutiérrez and Instituto Multidisciplinario de Investigacion en Patologias Pediatricas (IMIPP), CONICET-GCBA, Buenos Aires, Argentina; 2 Bayer AG, Research & Development, Clinical Development & Operations, Acute & Chronic Care & Pediatrics, Berlin, Germany; 3 Bayer AG, Clinical PK CV, Research & Development – Pharmaceuticals, Wuppertal, Germany; 4 Bayer AG, Pharmaceuticals, Chief Medical Office, CMO MA Sustainability, Wuppertal, Germany; 5 Department of Paediatrics, Schulich School of Medicine and Dentistry, Western University, London, Ontario, Canada; FDA: US Food and Drug Administration, UNITED STATES OF AMERICA

## Abstract

Nifurtimox has been used for over 50 years to treat patients with Chagas disease, a potentially life-threatening neglected tropical disease caused by the protozoan parasite *Trypanosoma cruzi*. Without effective antitrypanosomal treatment, the infection can persist and progress to a chronic, often debilitating, clinical form. Migration and urbanization, as well as the shifting distribution of the parasite’s insect vector, have contributed to the emergence of Chagas disease as a global health threat. Administration of nifurtimox involves adjusting the dose for age and body weight. Particularly for children, this often requires the previously available 120 mg tablet to be divided manually, which could be problematic. To address this challenge, a new formulation tablet of nifurtimox was developed. Available in two dose strengths, 30 mg and 120 mg, the new formulation tablets contain a functional score line to facilitate accurate division. In addition, the formulation now allows rapid and easy dispersion in water to form a slurry for use by patients with difficulty swallowing tablets. These features enable more accurate body-weight-based and age-appropriate dosing and administration, which should prove beneficial for younger patients, including newborns and babies with a body weight ≥2.5 kg. Development of the new formulation nifurtimox tablets was guided by substantially updating pharmacological and clinical knowledge of the drug to meet current standards and regulatory requirements. This was achieved by conducting a substantial array of additional non-clinical and clinical studies to better understand and characterize clinically relevant aspects of nifurtimox pharmacokinetics. The efficacy and safety of the new tablet in children with Chagas disease was subsequently demonstrated in a large prospective randomized clinical trial with prolonged follow-up. In the present paper, we review key findings that contributed to the successful clinical development of the new formulation nifurtimox tablet, the availability of which redefines the treatment of young patients with Chagas disease.

## Background

Chagas disease (American trypanosomiasis) is a potentially life-threatening neglected tropical disease caused by the parasite *Trypanosoma cruzi* [1–3]. In the natural course of the disease, there are two distinct clinical phases: acute and chronic [4,5]. In the acute phase, which lasts 4–8 weeks, approximately 90% of infected individuals have no or mild non-specific symptoms, and ~10% develop clinical symptoms, with severe disease (e.g., cardiac, digestive, and/or hematologic manifestations) occurring in up to half of those. The acute infection is fatal in 0.2–0.5% of cases [4].

In the majority of cases, acute phase symptoms resolve spontaneously without treatment, and the infection moves into a chronic phase. Around two-thirds of infected individuals continue with persistent asymptomatic disease for life (the indeterminate chronic phase of Chagas disease). Clinical symptoms, often debilitating, can develop in 30–40% of those with chronic Chagas disease 10–30 years after acquiring the infection [5]. Cardiomyopathy is the most serious manifestation of chronic Chagas disease, occurring in approximately 90% of symptomatic cases. Gastrointestinal, neurological, or mixed pathology disorders develop in 10% of patients; the chronic gastrointestinal manifestations, such as megacolon and megaesophagus, impact the quality of life of those affected and can cause life-threatening complications [6].

Diagnosis of Chagas disease is based on clinical observations, epidemiological information, and the results of parasitological, serological, and molecular laboratory tests [3,7]. Microscopy of blood films, microhematocrit test (in which the rapid movements of the flagellated trypomastigote form of the parasite are visible by microscopy of peripheral blood), and polymerase chain reaction (PCR) testing for *T. cruzi* deoxyribonucleic acid (DNA) are used to diagnose suspected acute infection.. As the infection progresses into the chronic phase, parasitemia declines to become very low or intermittent as the trypomastigotes invade nucleated cells in tissues. PCR may be used to establish the parasitic load, but a negative PCR test does not exclude chronic *T. cruzi* infection. A variety of *T. cruzi* antigens trigger a strong antibody response to the parasite and result in seropositivity with high titres of IgG anti-*T. cruzi* antibodies. Serological tests for anti-*T. cruzi* antibodies, such as enzyme-linked immunosorbent assay (ELISA), indirect immunofluorescence, or indirect hemagglutination, are used for the primary diagnosis of chronic Chagas disease but are not useful in suspected acute disease [8,9]. The use of at least two serological tests that are based on different methodological principles are recommended, and a third test should be used if only one of the two initial tests is positive.

The most common route of infection by *T. cruzi* in endemic countries is via the triatomine insect vector, but other routes of infection, notably congenital transmission, are also important. In areas where the vector is controlled or absent, the relative importance of mother-to-child transmission increases [10]. An oral route of infection by *T. cruzi* via contaminated food and drink products has also been highlighted recently [11,12].

### The growing burden of Chagas disease

Chagas disease confers a substantial medical, social, and economic burden [3]. Global estimates indicate that in 2019, 6–7 million individuals were infected with *T. cruzi*, amounting to a disease-associated burden of 275,377 disability-adjusted life-years; a further ~75 million people were at risk of infection [8]. In the Americas, the disease has an average annual incidence of 30,000 new cases, causing 12,000 deaths per year, of which approximately 9,000 newborns are infected during gestation [9]. Outside of Latin America, the USA has the highest estimated burden of Chagas disease with around 288,000 infected persons in the period 2014–2018 [13], increasing from 240,000 cases in 2012 [14]. Designation of Chagas disease as a neglected tropical disease by the World Health Organization (WHO) in 2005 has promoted recognition of its disease burden; however, in many countries, systematic screening and diagnostic testing for Chagas disease have not been implemented [2]. Analysis of the Global Burden of Disease Study data has revealed the age-standardized prevalence rates per 100,000 population in 2019 to be 4.12 in Europe, 15.55 in North America, and 933.76 in Latin America [15].

With human migration, international travel, climate change, and other factors contributing to a dynamic epidemiology, the global distribution of Chagas disease is changing. In Latin America, Chagas disease remains endemic in 21 countries, but urbanization has shifted the occurrence of the disease from rural to urban environments [5,9]. Migration has also contributed to an increase in the number of cases in non-endemic countries, including Italy, Spain, USA, Canada, Japan, and Australia [5]. The prevalence rate of the disease in Europe increased by 111% from 1990 to 2019, with immigration of *T. cruzi*-infected Latin Americans contributing to substantial increases in some countries, such as Spain (+434%), Italy (+177%), and the UK (+88%) [15]. Changes in triatomine bug distributions, partly as a result of climate change, have been reported, although increasing and decreasing trends have been shown in different parts of the world [16,17]. Chagas disease can thus be considered both a persistent and emerging infectious disease, with non-vectorial routes of infection, particularly mother-to-child transmission, becoming increasingly important in many non-endemic regions [3].

The WHO has targeted the elimination of transmission of *T. cruzi* infection to humans as a public health goal, while acknowledging that the existence of a widespread reservoir of *T. cruzi* in wild animals precludes eradication of the infection. Achieving this goal requires substantial reductions in transmission by the known routes and increased access to diagnosis and antiparasitic treatments [2].

## Treatment of Chagas disease

Early diagnosis and treatment of Chagas disease increase the chances of cure, reduce the risks of disease progression and the development of complications in infected patients, and prevent congenital transmission or transmission through blood or organ donation [1–3].

The current treatment options for Chagas disease are limited to benznidazole and nifurtimox. Both drugs are trypanocidal and have been shown to clear parasitemia and improve disease symptoms when patients are treated early in the course of the infection [3,18]. The recommended treatment regimen for acute and chronic Chagas disease is dosing two (benznidazole) or three (nifurtimox) times per day for at least 60 days. However, the availability of these medications in some countries has, at times, been erratic and impacted by national (and sometimes regional) bureaucratic procedures/requirements, among other factors [19]. Restricted or variable availability of antitrypanosomal drugs represents a clear barrier to treatment for those with *T. cruzi* infection [20].

Response to treatment is reflected by a decline in antibody response to *T. cruzi* and measured by conventional serological tests. In acute Chagas disease, antitrypanosomal treatments achieve high cure rates, and the antibody response to *T. cruzi* becomes negative relatively soon after treatment. In the chronic phase of the disease, titres of anti-*T. cruzi* antibodies decline slowly, and confirmation of cure by conversion to seronegative may take decades after treatment. Published rates of seronegative conversion measured post-treatment by conventional serological tests range from 5% after 5 to 10 years to 45% after more than 20 years [21].

Searches for alternative biomarkers of Chagas disease have identified a variety of parasite- and host-derived factors that may correlate with disease progression or treatment response [22–24]. For example, antibodies to the *T. cruzi* protein F29 have been shown to disappear earlier than those measured by conventional serology in children treated with nifurtimox [25]. Moreover, a positive quantitative PCR (qPCR) test for *T. cruzi* DNA is widely considered as evidence for therapeutic failure. This test is also accepted in some countries as an alternative to direct parasitological tests for the diagnosis of *T. cruzi* infection in the acute phase or in reactivated or congenitally-acquired Chagas disease [22]. However, there is currently no reliable biomarker of parasitological cure in chronic Chagas disease, nor any biomarker that has been shown to predict the risk for long-term complications such as cardiac or gastrointestinal dysfunction [26]. All potential biomarkers proposed so far still require validating and standardizing, and none have attracted the regulatory approval or commercial interest required to progress towards integration into the standard diagnostic process. Thus, the current standard criterion for cure accepted by health authorities remains seronegative conversion measured by ≥2 different types of conventional serological assay. This criterion, while widely accepted, suffers from a number of drawbacks, notably the fact that a large proportion of patients with chronic *T. cruzi* infection do not achieve, or require decades to achieve, seronegative conversion; there are also no studies directly correlating this antibody response to reductions in complications. Of note, the United States Food & Drug Administration (US FDA) has accepted a surrogate marker of ≥20% decrease in optical density in two conventional ELISA tests (but not necessarily seronegative conversion) for anti-*T. cruzi* antibodies at 1-year post-treatment as a pharmacodynamic endpoint that can be evaluated by the same ELISA tests within a reasonable amount of time after treatment to verify response [26, 27].

## Nifurtimox

Nifurtimox, an antiparasitic nitrofuran, has been used to treat Chagas disease for over 50 years [18]. Following clinical studies that demonstrated its efficacy and safety, the drug was approved for clinical use in several Latin American countries in the 1970s [28], where it became available as the commercial product Lampit. Recognition of nifurtimox as an effective treatment for this neglected tropical disease merited its inclusion in the first WHO Model List of Essential Medicines in 1977, where it has remained since.

When originally developed, evaluation of nifurtimox for clinical use was not conducted according to the same epidemiological context and levels of medical requirements of today. In response to the evolving epidemiology of Chagas disease, Bayer Pharmaceuticals, the originator of nifurtimox, recently undertook a development program to support a new tablet formulation of the drug. The program included a series of regulatory-compliant studies to update and improve the pharmacology, efficacy, and safety information about the compound. The findings of these studies supported registration of the new tablet formulation in two dose strengths (30 mg and 120 mg) in various countries, e.g., Germany, Spain, the USA, and some Latin American countries, where the spread of Chagas disease drives a growing need for effective antitrypanosomal treatment.

### Methods

This article reviews the research that underpins the successful development of the new tablet formulation of nifurtimox, thereby facilitating appropriate dosing of this antitrypanosomal treatment to patients with Chagas disease aged from birth to 18 years.

### A better understanding of nifurtimox clinical pharmacology

Until recently, knowledge of the pharmacokinetics of nifurtimox and its absorption, distribution, metabolism, and excretion profile in humans and model mammalian systems relied to a large extent on studies conducted many years ago that are now outdated [29–34]. As shown previously, the bioactivation of nifurtimox is mediated by type I nitroreductase enzymes [35,36]. Although satisfactory for the drug approval process at the time, such studies were conducted using techniques that have been superseded and now appear incomplete or insufficient to meet the current standards of pharmacological investigation and regulatory requirements. In particular, the limited investigation of drug disposition and pharmacokinetics in past studies prevented the evaluation and prediction of drug–drug interactions or the impact of organ failure (e.g., kidney or liver failure) on nifurtimox pharmacokinetics. The development program for the new formulation tablet therefore included a series of phase 1 clinical studies to fill the gaps in our understanding of nifurtimox clinical pharmacology.

### Nifurtimox pharmacokinetics

The recent phase 1 studies confirmed that, after oral administration, nifurtimox is rapidly absorbed and eliminated; the time from administration to maximum plasma drug concentration is 2–3 hours, and the average elimination half-life is approximately 3 hours [37,38]. When four of the new formulation 30 mg tablets were given at the same time (i.e., a 120 mg dose) to patients with Chagas disease after a high-fat, high-calorie meal, systemic exposure was increased by ~71% compared with the same dose given to patients in a fasted state; smaller increases in exposures occurred with low-fat or dairy-based meals. Nifurtimox should, therefore, be taken with food, which maximizes exposure for the given dose as well as improve gastrointestinal tolerability. Furthermore, the time to reach maximum plasma concentration (Tmax) increased by approximately 1 hour if the patient had eaten before dosing.

The effect of food on nifurtimox pharmacokinetics is likely to be less marked in patients with Chagas disease. In real world settings, the routine diet of these patients is unlikely to include high-fat meals such as those recommended by the FDA for use in food-effect bioavailability and fed bioequivalence studies [39]. Clinical experience with nifurtimox has shown that treatment is effective in children and adolescents whose diets, as a group, are comprised of a variety of food stuffs [40]. For routine therapeutic use of nifurtimox, however, dosing regimens do not have to be adjusted according to diet [38]. In addition, as demonstrated by *in vitro* investigations, the absence of cytochrome P-450 enzyme or drug transporter-mediated pharmacokinetic interactions for nifurtimox or its two most abundant metabolites (designated M-6 or M-4) [41] provides reassurance for the safety and tolerability of other medicines used by patients with Chagas disease receiving nifurtimox treatment.

### Biotransformation and elimination of nifurtimox

Although early *in vivo* studies established that the metabolism of nifurtimox was extensive [29,30], the fate of the drug and the pathways and enzymes involved in its metabolism remained largely unknown until recent investigations using contemporary high-resolution laboratory techniques revealed important aspects of the complex biotransformation of nifurtimox [41,42] (Box 1).

**Box 1. Some key features of nifurtimox metabolism and excretion**

**Fig 1:**
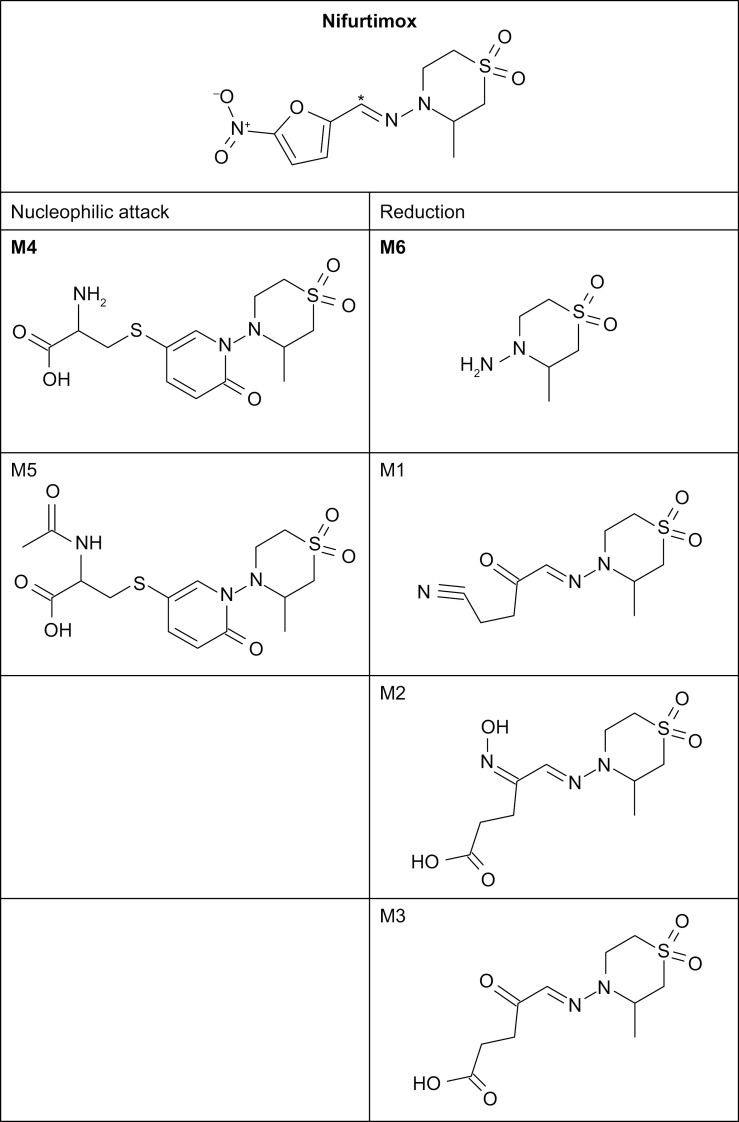
Main metabolic pathways and resultant metabolites of nifurtimox [42, 43]. According to previous investigations, M-6 and M-4 were the only metabolites (among those quantified), that showed relevant plasma exposure in humans and exceeded or approached the guideline threshold of 10% of total exposure [42,44,45]. Please see the marketing authorization for further safety and drug–drug interaction testing [42,46]. *Indicates position of ^14^C label.

- *In vivo* biotransformation of nifurtimox yields more than 30 metabolites, and molecular structures have been provisionally identified for the majority of them (**Fig 1**).
- Metabolites M-1 to M-6 are the most abundant. Of these, M-6 and M-4 are the only metabolites that achieve relevant exposure levels in patients, and studies in rats showed no concerns for the potential toxicity of these metabolites during the use of nifurtimox at therapeutic doses.
- Nifurtimox is metabolized mainly by reduction or nucleophilic attack, with some oxidation.
- The absence of involvement of the typical drug-metabolizing liver and kidney enzymes in nifurtimox biotransformation is consistent with the lack of effect on the main cytochrome P-450 enzyme and drug transporters, suggesting a low risk of metabolic drug–drug interactions.
- Based on animal studies, the main routes of excretion of nifurtimox in humans are likely via the urine and feces.
- By mass balance study, the fate of nifurtimox after oral administration has not been fully elucidated; approximately 50% of the administered dose is excreted in urine as metabolites, with only trace amounts of the unchanged drug.

### Nifurtimox dosing and administration

For many patients, achieving an approximate weight-adjusted dose of nifurtimox required the previously marketed 120 mg tablet to be divided. The need for tablet subdivision is especially common for children. As a result, the dosing regimen and tablet format presented barriers to accurate and consistent dosing as well as treatment adherence. In real-world settings, regular administration of the medication is often the responsibility of parents or others who lack medical training and have limited resources [38]. In addition, for young children, infants, and adults with dysphagia who have difficulty swallowing solids, there is an increased risk of aspiration of the whole tablet or smaller fragments. For such patients, administration involved the tablet being crushed and mixed with a small amount of liquid or food, which adds to the uncertainty regarding the accuracy and content uniformity of the prepared dose and the subsequent pharmacokinetics of the drug in the dose administered.

Across clinical practice, tablet splitting for dose adjustment is common, particularly for pediatric patients, but the potential lack of uniformity among the resultant half tablets raises clinical risks [47]. The inclusion of score lines are acknowledged as key to permit easy and accurate subdivision of a tablet with minimal loss of mass [48,49]. Further, for young children with Chagas disease (or another neglected tropical disease), the most suitable age-appropriate formulations for optimal treatment efficacy and safety are likely to be dispersible tablets or multiparticulate formulations [50].

### The new formulation tablet: biopharmaceutical characterization and consequences for dosing and administration

**Fig 2:**
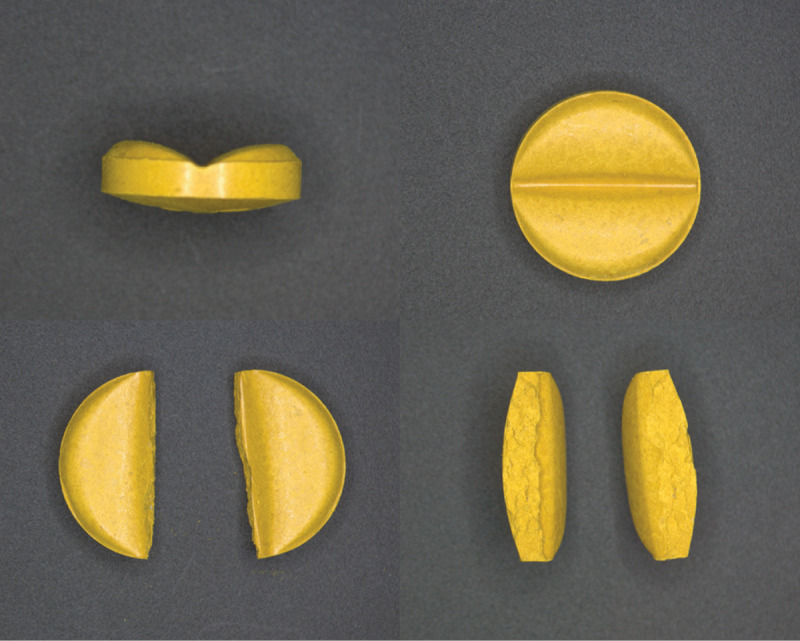
The format of the new formulation nifurtimox tablets (upper images show whole 30 mg tablets) allows each 30 mg tablet to be snapped reproducibly into two equal fragments (lower images) [Images from Bayer AG]. Reprinted from Stass H, Just S, Weimann B, Ince I, Willmann S, Feleder E, et al. Eur J Pharm Sci. 2021;166:105940. https://doi.org/10.1016/j.ejps.2021.105940. Copyright 2021, reproduced with permission of Heino Stass [38].

To aid the preparation of different nifurtimox doses, easily divisible tablets were developed in dose strengths of 30 mg and 120 mg based on the granules of the existing immediate-release formulation (**Fig 2**) [38]. Containing the same excipients as the existing clinical formulation, the new formulation was obtained by adjusting the granule properties and residual moisture content of the tablets during the manufacturing process [38]. For both dose strengths, a functional score line was incorporated to facilitate division of the tablets [49]. With this design feature, 30 mg and 120 mg tablets can be divided along score lines to give two equal fragments containing defined amounts of the active ingredient (i.e., 15 mg and 60 mg of nifurtimox, respectively). Splitting the tablets can be done easily by hand and does not require any special tool or splitting device. Overall, the new formulation and design allow administration of smaller and more accurate dose increments than was previously possible. The tablets were also formulated to disintegrate quickly in a small quantity of water, e.g., one-half teaspoon (2.5 mL), to form a suspension of fine particles (a slurry), which can be administered to patients unable to swallow tablets. The new tablet formulation of nifurtimox gained full FDA approval in 2023 [51].

The recently reported phase 1 clinical studies provided important characterization of the biopharmaceutical properties of the new tablet formulation of nifurtimox. Two pharmacokinetic studies in adult patients with Chagas disease compared the biopharmaceutical features of the new formulation 30 mg tablet with that of the existing 120 mg tablet [37]: one study demonstrated that four 30 mg tablets of the new nifurtimox formulation were bioequivalent to one existing 120 mg tablet; the other study confirmed that the bioavailability of the new 30 mg formulation was unaffected whether administered in tablet form or as an aqueous slurry. In a separate study [38], the profile of nifurtimox dissolution from the new tablet formulation was confirmed to be compliant with the specification set in regulatory guidance [52, 53], and the bioavailability of nifurtimox was shown to be unaffected by tablet dissolution rates, which might vary among production batches.

**Fig 3:**
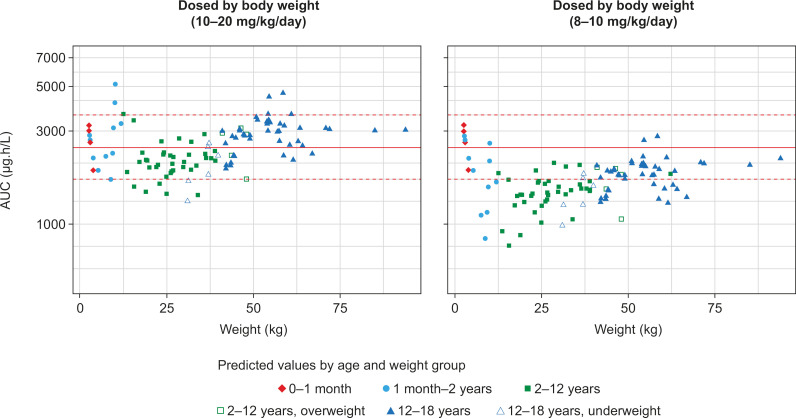
Estimated pediatric exposure to nifurtimox based on body weight adjustment using single-dose ranges (10–20 mg/kg/day and 8–10 mg/kg/day). Dotted redlines show the 5th (1688 μg.h/L) and 95th (3573 μg.h/L) percentiles. Solid red lines show the median (2441 μg.h/L) of plasma nifurtimox exposure in adults scaled based on a 500 mg daily dose. AUC, area under the curve. Reprinted from Stass H, Ince I, Grossmann U, Weimann B, Willmann S. AAPS J. 2022;24(5):92. https://doi.org/10.1208/s12248-022-00742-w. Copyright2022, under license CC-BY 4.0 and with permission of Springer Nature [54].

From the results of population pharmacokinetic (popPK) modeling, the recommended dosing using the new tablet formulation – in regimens of 10–20 mg/kg/day for patients <12 years of age with body weight <40 kg and 8–10 mg/kg/day for patients ≥12 years of age with body weight ≥40 kg – achieves exposures to nifurtimox within the range that has been shown to be effective in treating adults [54]. These regimens are unaffected by sex and are dose-linear across the pediatric dose range [55]. Moreover, popPK modeling of these two standard pediatric dose ranges showed that in children younger than 12 years, exposure tends to increase sharply as body weight increases. Switching from the higher to lower range at thresholds of age 12 years or body weight 40 kg smooths the changes out in exposure with increasing age and reduces the likelihood of overexposure in adolescents (**Fig 3**). It was noted that further refinement of the dosing regimen using an additional dose threshold and more dose ranges would likely smooth out the relationship between exposure and age/body weight even more, but such improvements would not outweigh the risks arising from increased complexity of administration, such as increased non-compliance or potential dosing errors [54]. Conversely, simplifying the regimen would likely result in greater deviation from target exposure ranges in some patients. However, no relationship is apparent between the level of exposure to nifurtimox and serological measures associated with parasite clearance, or between exposure and the occurrence/severity of typical treatment-emergent adverse events (TEAEs) [54].

### Clinical trials of the new formulation nifurtimox tablet: CHICO and CHICO SECURE

A pivotal step towards the regulatory approval of the new tablet formulation of nifurtimox was the demonstration of its clinical effectiveness in the pediatric population in a large randomized clinical trial with prolonged follow-up. The **CH**agas disease **I**n **C**hildren treated with nifurtim**O**x (CHICO) study was a prospective, historically controlled, phase 3 clinical trial to evaluate the efficacy and safety of nifurtimox in children with Chagas disease [40]. Children aged from birth to <18 years were randomly assigned 2:1 to nifurtimox treatment for 60 days or 30 days. The primary outcome was the anti-*T. cruzi* serological response (seronegative conversion or ≥20% seroreduction in mean optical density from baseline determined by two conventional ELISAs) at 12 months in the 60-day treatment group versus historical placebo-treated controls. The shorter treatment arm was included for exploratory purposes and to allow comparison of the treatment response rates in a secondary analysis of the primary outcome.

**Fig 4:**
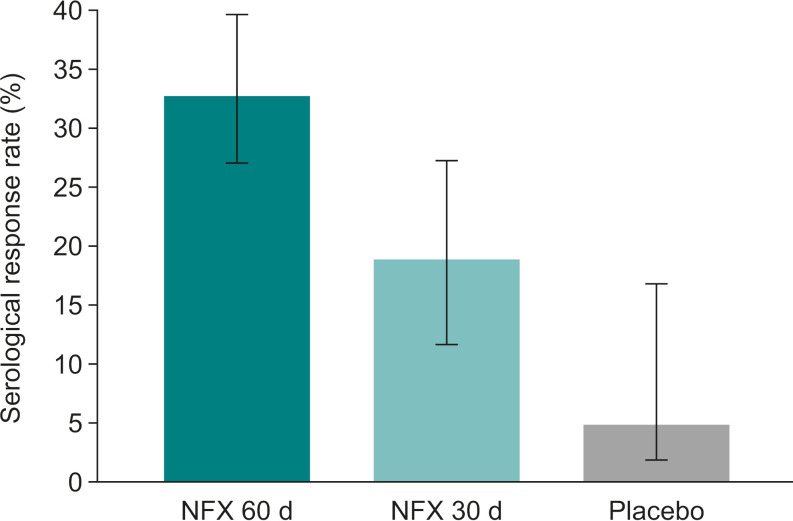
Serological response rates (95% CI) to 60-day and 30-day nifurtimox treatment assessed by conventional serological testing 12 months after the end of treatment [40]. Response was defined as seroreduction (in patients aged ≥8 months to <18 years at randomization) or seronegative conversion (in all patients). Seroreduction was defined as at least a 20% reduction in mean optical density measured by two conventional ELISA tests, and seronegative conversion was defined as a negative anti-*T. cruzi* IgG concentration by two conventional ELISA tests. For placebo, the clinical response rate (95% CI) was derived from a published study [56]. CI, confidence interval; ELISA, enzyme-linked immunosorbent assay; IgG, immunoglobulin G; NFX, nifurtimox.

The CHICO study confirmed the efficacy of age- and weight-adjusted 60-day treatment with nifurtimox compared with historical placebo treatment at 12 months. Nifurtimox for 60 days resulted in seronegative conversion (n=10) and seroreduction (n=62) in 72 patients. This corresponded to an overall serological response in 32.9% (95% confidence interval [CI] 26.4%, 39.3%) of treated patients and confirmed the superiority of treatment relative to the upper 95% CI of 16% in the placebo group (**Fig 4**). With a corresponding response rate of 18.9% (95% CI 11.2%, 26.7%), the efficacy of the 30-day nifurtimox regimen was not demonstrated based on the pre-planned per-protocol analysis. The difference in overall treatment response between the two regimens at 1-year follow-up was 14.0% (95% CI 3.7%, 24.2%).

In patients aged 2 to <18 years, the 60-day regimen was more effective than the 30-day regimen. The serological response was stronger in children younger than 2 years, and the serological and parasitological response rates in this age group appeared to be similar across the two treatment regimens; however, the study included only a few patients in this age group and was not powered to support formal statistical comparison of the two regimens.

Treatment was well tolerated, with mostly mild or moderate adverse events (AEs) and a low incidence of discontinuation. Less than 30% of TEAEs were considered to be treatment-related. In patients treated with nifurtimox for 60 days, the most common TEAEs were: vomiting, 14.6%; abdominal pain or upper abdominal pain, 13.2%; headache, 12.8%; and decreased appetite, 10.5% [51]. The majority of TEAEs were mild to moderate in severity and resolved upon stopping treatment.

**Fig 5:**
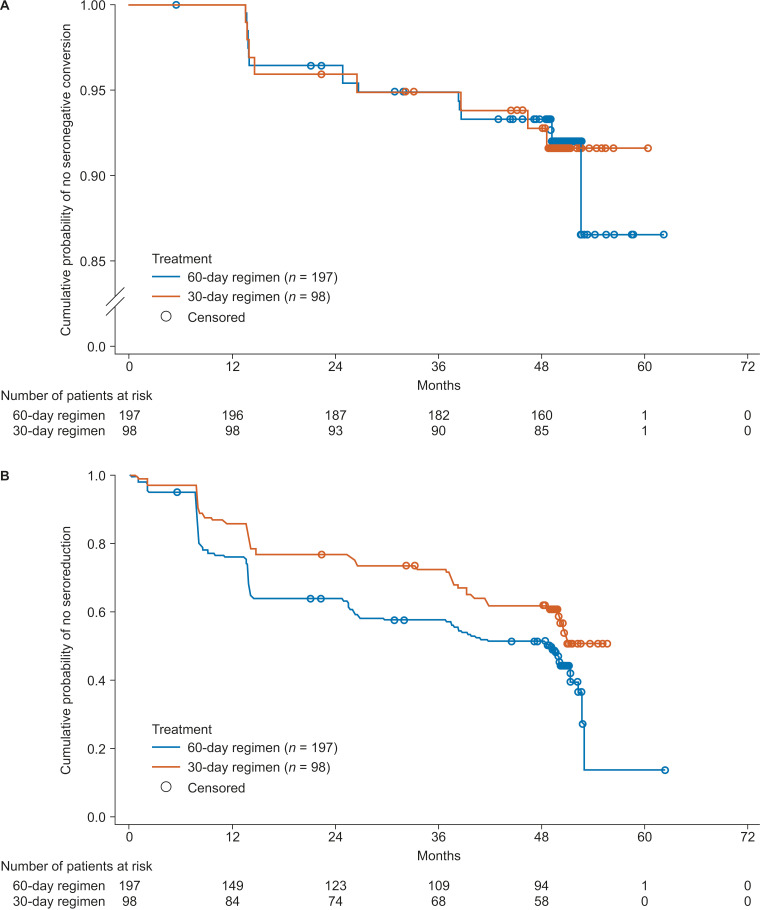
Kaplan–Meier curves of (A) seronegative conversion(B) ≥20% to 100% seroreduction in patients receiving 60-day or 30-day nifurtimox treatment regimens (full analysis set, N=295). Patients who received other antitrypanosomal treatments were considered censored. Serological responses were measured by recombinant enzyme-linked immunosorbent assay and indirect hemagglutination assay, and negative results for both tests were required for the patient to be considered to have achieved seronegative conversion. Reprinted from Altcheh J, Sierra V, Ramirez T, Pinto Rocha JJ, Grossmann U, Huang E, et al. Antimicrob Agents Chemother. 2023;67(4):e0119322. https://doi.org/10.1128/aac.01193-22. Copyright 2023, under license CC-BY 4.0 and with permission of the American Society for Microbiology [57].

The **CHICO** follow-up for **SE**roconversion and **CURE** (CHICO SECURE) study [57] evaluated seronegative conversion in patients who were followed up for 4 years after the end of nifurtimox treatment in the CHICO study. The CHICO SECURE study showed that the number of cases with seronegative conversion increased during the 4-year post-treatment follow-up (**Fig 5A**). In patients who were treated for 60 days, seronegative conversion was observed in seven (3.55%), three (1.52%), three (1.52%), and three (1.52%) patients at the 1-, 2-, 3-, and 4-year post-treatment follow-up assessments, respectively. In those treated for 30 days, seronegative conversion at the corresponding follow-up time points was documented in four (4.08%), one (1.02%), two (2.04%), and one (1.02%) patient(s), respectively. At the end of the study, the seronegative conversion rate was 7.11% in the 60-day nifurtimox regimen and 6.12% in the 30-day regimen. In addition, the proportion of patients who tested negative for *T. cruzi* DNA by qPCR increased from approximately 47% at baseline to more than 90% in both treatment groups by 1-year post-treatment. Nearly all patients who became *T. cruzi* qPCR-negative showed successive negative results in up to 10 consecutive *T. cruzi* qPCR tests; positive qPCR results were recorded for only eight patients at any post-treatment follow-up visit. Progressive seroreduction was seen in both treatment groups (**Fig 5B**). At the 4-year follow-up point, ≥20% seroreduction was observed in around 50% and 40% of the 60- and 30-day treatment groups, respectively. No AEs considered possibly related to treatment or to protocol-required procedures emerged during post-treatment follow-up.

Exploratory analysis of the serological response by age group found that children aged <2 years at baseline were more likely to reach seronegative conversion at 4-year follow-up than older children with both treatment regimens. In this age group, seronegative conversion was observed in 13 of 30 patients (43.33%) in the 60-day regimen and 7 of 13 patients (53.85%) in the 30-day regimen. As a prolonged follow-up phase of the CHICO trial, the CHICO SECURE study was not designed or powered to compare the two treatment regimens, so these results should be interpreted with caution. Similarly positive outcomes in adequately powered studies are needed to confirm the potential efficacy of the shorter treatment regimen, particularly in the youngest age group, that was observed in the CHICO SECURE study.

## Nifurtimox for pediatric Chagas disease: evidence from real world use

Several recently reported observational studies have provided additional support for the efficacy of nifurtimox in children with Chagas disease. The importance of treating *T. cruzi*-infected patients during childhood was emphasized by the results of a retrospective cohort study of patients (n=289) treated with nifurtimox between 1980 and 2019 (i.e., using the originally marketed 120 mg tablets) [58]. This study included 199 children, approximately 42% of whom had congenitally transmitted infection, treated with a median (interquartile range [IQR]) nifurtimox dose of 11 (10–12) mg/kg/day divided into two (n=139) or three doses (n=60) for a median (IQR) duration of 62 (60.5–73) days. After a median (IQR) follow-up of 37.7 (12.2–85.3) months of patients with positive serology at baseline (n=187), seroconversion occurred in 95 children (50.8%) and seroreduction (≥20% decrease in anti-*T. cruzi* antibodies from baseline) in 141 children (75.4%). Seroconversion in response to treatment occurred earlier and was more probable in younger patients, with the probability of seroreduction also increasing with younger age. Treatment was associated with improvement of clinical symptoms, if present, in all patients. In another descriptive study, long-term cardiology outcomes were assessed in children who had received benznidazole or nifurtimox for 60–90 days since *T. cruzi* diagnosis with a median (range) follow-up time of 10 (6–20) years. During the follow-up period, only four out of 234 patients showed electrocardiogram alterations probably related to Chagas disease; none of them were treated with nifurtimox [59].

In support of the safety results reported in the CHICO clinical trial, a large retrospective medical records-based study conducted at the Ricardo Gutiérrez Children’s hospital in Argentina demonstrated a similar safety and tolerability profile of nifurtimox in children treated for 60–90 days [60]. AEs and TEAEs were less frequent and occurred later after the start of treatment in children than in adults; they were rarely a cause of treatment discontinuation. A separate review of long-term follow-up data at a regional reference center in Santiago del Estero, Argentina, also identified a lower frequency of nifurtimox-associated AEs in children during treatment for Chagas disease in both the acute (n = 1490) and chronic (n = 466) phase compared with adults (n = 71 and n = 968, respectively) [61].

The lower frequency of AEs with nifurtimox treatment versus benznidazole was also noted in a recent systematic review and meta-analysis of studies evaluating the effects of antitrypanosomal treatment in female patients with Chagas disease of childbearing age [62]. This analysis highlighted that transmission of Chagas disease from mother to child can be prevented (odds ratio 0.05; 95% CI: 0.01–0.27; *P* = 0.000432; I2 = 0%) with both treatments [62]. Currently, the labels for both nifurtimox and benznidazole warn against their use in pregnant women due to the potential risk for the fetus [51,63].

## Towards shorter treatment regimens

The duration of treatment, the frequency of administration, and the occurrence of AEs are likely factors contributing to poor compliance with trypanocidal medication regimens [64–66]. Treatment durations shorter than 60 days for benznidazole have been proposed to improve compliance and tolerability based on the double-blind, Phase 2 BENDITA study [67], but clinical study reports of short-term treatment are scarce. A retrospective medical records-based study in Argentina, for example, found that nifurtimox was effective in preventing congenital transmission of Chagas disease in all 17 mothers, including one 14-year-old girl, who received treatment for ≤30 days before pregnancy [68] (note: according to national clinical practice guidelines in Argentina [69], the recommended antitrypanosomal treatment duration is 60 days, but a treatment regimen of 30 days is considered acceptable as the minimum duration for selected patients. In Bolivia, antitrypanosomal treatment for 30 days is recommended for children with congenitally-acquired *T. cruzi* infection [70]). Nifurtimox treatment is well tolerated in children and less likely to be discontinued due to AEs than in adults [40,60]; the paramount consideration, however, is whether comparable efficacy is preserved with a shorter treatment. Further studies with adequate statistical power and an appropriate duration of follow-up, particularly in children aged <2 years at the time of diagnosis, are needed to evaluate and statistically demonstrate the serological and parasitological efficacy and safety of shorter nifurtimox treatment durations than the 60-day FDA-approved regimen.

## Conclusions

Chagas disease remains an important parasitic disease in areas of long-term endemicity and is emerging as a serious and growing healthcare concern elsewhere. Approved treatment options for patients with Chagas disease are still limited to two trypanocidal agents, nifurtimox and benznidazole. The availability of nifurtimox in the new formulation easily divisible, dispersible tablets in two dose strengths (30 mg and 120 mg) facilitates its administration in patients with Chagas disease of all age groups and body weight.

Recent studies using contemporary analytical techniques to investigate the pharmacokinetics of nifurtimox in humans have improved our understanding of the disposition and complex metabolic fate of this drug as well as the products of its biotransformation. Conducted to support the regulatory assessment of the new formulation nifurtimox tablet, these studies have also provided reassurance regarding the absence of effects from exposure to nifurtimox metabolites and the low risk of drug–drug interactions during its therapeutic use. Notably, an important dosing consideration confirmed by these recent studies is that nifurtimox should be taken with food to maximize exposure.

The results of a large prospective randomized clinical trial (CHICO) confirmed the importance of prompt effective treatment following early diagnosis and the appropriateness of an age- and weight-adjusted nifurtimox regimen for pediatric patients with Chagas disease. Based on the clinical evidence, treatment with nifurtimox for 60 days should be preferred over 30 days for patients aged 2 to 18 years as a greater proportion achieved ≥20% to 100% seronegative conversion with longer treatment. In a small subgroup of children younger than 2 years, 60-day and 30-day duration of treatment appeared to be equally efficacious. Nifurtimox was well tolerated overall. There was a tendency of more frequent AEs across the various age groups with longer than shorter treatment, and discontinuation was reported in 5.5% and 1.8% of patients, respectively. However, study drug-related AEs occurred in a similar proportion of patients treated for either 60 days or 30 days. Of note, the frequency of AEs was lower in younger patients for both treatment durations, especially in newborn patients [40]. The CHICO study also provided valuable information regarding the biomarkers of treatment response and the potential use of a shorter regimen in pediatric patients that could guide the direction of future research.

The availability of the new formulation tablet of nifurtimox allows patients with Chagas disease in Latin America, the USA, and elsewhere in the world to have access to an approved treatment from birth. The successful development and approval of this age-appropriate tablet formulation create the opportunity for early effective therapy in more *T. cruzi*-infected children, particularly newborns and young infants likely to have acquired the infection congenitally. Encouraging timely detection and treatment of Chagas disease would bring important benefits, such as reducing the risks of serious complications later in life for patients with chronic disease and, for females destined to reach child-bearing age, breaking the cycle of maternal-fetal *T. cruzi* transmission.

The new tablet formulation of nifurtimox redefines the treatment of young patients, particularly those aged under 2 years, with this neglected tropical disease; one that is gaining global importance by emerging into new territories while persisting in historically endemic areas.

**Key Learning Points**

- Nifurtimox is now available in a new formulation tablet in dose strengths of 30 mg and 120 mg.
- The new formulation nifurtimox tablets are easily and accurately divisible to facilitate preparation of dose appropriate to the patient’s age and body weight.
- These tablets are dispersible in water to allow administration to patients at risk of aspirating whole or part solid tablets.
- The successful development and US Food and Drug Administration (FDA) approval of the new tablets is the outcome of an extensive contemporary series of preclinical and clinical studies that have enhanced knowledge of nifurtimox as a compound and in this novel formulation.
- Improved understanding of the fate of nifurtimox after oral administration, its resulting metabolite profile, and the potential for drug–drug interactions attributable to the drug or its metabolites supports a favorable risk-benefit assessment.

**Five Key Papers**

- Altcheh J, Castro L, Dib JC, Grossmann U, Huang E, Moscatelli G, et al.; CHICO Study Group. Prospective, historically controlled study to evaluate the efficacy and safety of a new pediatric formulation of nifurtimox in children aged 0 to 17 years with Chagas disease one year after treatment (CHICO). PLoS Negl Trop Dis. 2021;15(1):e0008912. https://doi.org/10.1371/journal.pntd.0008912.
- Altcheh J, Sierra V, Ramirez T, Pinto Rocha JJ, Grossmann U, Huang E, et al. Efficacy and safety of nifurtimox in pediatric patients with Chagas disease: results at 4-year follow-up in a prospective, historically controlled study (CHICO SECURE). Antimicrob Agents Chemother. 2023;67(4):e0119322. https://doi.org/10.1128/aac.01193-22.
- Stass H, Just S, Weimann B, Ince I, Willmann S, Feleder E, et al. Clinical investigation of the biopharmaceutical characteristics of nifurtimox tablets – implications for quality control and application. Eur J Pharm Sci. 2021;166:105940. https://doi.org/10.1016/j.ejps.2021.105940.
- Stass H, Feleder E, Garcia-Bournissen F, Nagelschmitz J, Weimann B, Yerino G, et al. Biopharmaceutical characteristics of nifurtimox tablets for age- and body weight-adjusted dosing in patients with Chagas disease. Clin Pharmacol Drug Dev. 2021;10(5):542-555. https://doi.org/10.1002/cpdd.871.
- Stass H, Ince I, Grossmann U, Weimann B, Willmann S. Nifurtimox for treatment of Chagas disease in pediatric patients: the challenges of applying pharmacokinetic-pharmacodynamic principles to dose finding. AAPS J. 2022;24(5):92. https://doi.org/10.1208/s12248-022-00742-w.

## Data reporting

The authors confirm that all data underlying the findings of the CHICO and CHICO SECURE studies are fully available without restriction. All data files are available from the ClinicalTrials.gov website (trial identifier NCT02625974). For the availability of other data, please refer to the original publications detailed in the Five Key Papers.
